# Motivations and experiences of volunteers and patients in mental health befriending: a thematic analysis

**DOI:** 10.1186/s12888-019-2102-y

**Published:** 2019-04-17

**Authors:** Megan Cassidy, Rose Thompson, Rawda El-Nagib, Lauren M. Hickling, Stefan Priebe

**Affiliations:** 1 Unit for Social and Community Psychiatry, WHO Collaborating Centre for Mental Health Services Development, Newham Centre for Mental Health, Glen Road, London, E13 8SP UK; 2 McPin Foundation, 32-36 Loman St, London, SE1 0EH UK

**Keywords:** Befriending, Mental health, Volunteering, Motivation, Experience

## Abstract

**Background:**

Volunteers frequently participate in befriending schemes with people with mental illness. This study aimed to explore the motivations and experiences of volunteer befrienders engaging in these schemes in addition to the experiences of befriending recipients.

**Methods:**

Semi-structured interviews were conducted with 38 volunteers and 23 befriending recipients, across 12 mental health befriending schemes in the UK, and analysed using Thematic Analysis. Volunteers highlighted their motivations for wanting to befriend. Individuals discussed their experiences, including the benefits and any challenges.

**Results:**

Analysis of interviews revealed the motivations for individuals to volunteer in mental health care, the experiences of both volunteers and recipients of befriending, as well as how complex the role of befriender is. The three overarching themes were (1) Personal growth & altruism as motivations for volunteering, (2) Impact of “doing things” versus “being there” and (3) Negotiating between professional role and friendship.

**Conclusions:**

A number of personal and altruistic factors motivate individuals to volunteer in mental health care. The experiences of both volunteers and befriendees convey important factors affecting these relationships. In particular, the nuance of the befriending role and the ways in which it can impact the lives of recipients. Indeed, such factors need to be considered when formulating these befriending schemes.

## Background

Many people living with mental health problems experience difficulty in not only creating, but maintaining, meaningful social connections [8]. This difficulty often means that such individuals tend to have far fewer social networks than those without mental illness [3]; social networks that are, more often than not, restricted to other peers with mental illness, family members and mental health professionals [2, 6]. Thus, a number of the population living with mental illness experience an inability to integrate into their community [8] in addition to a profound feeling of social isolation [9]. Such social consequences are known to be related to poor health outcomes [11] and may ultimately create a barrier in the path to recovery (e.g. [23]).

Across the United Kingdom, and internationally, a number of initiatives have been put into place, both by health care providers and voluntary organisations to address this social isolation. Such attempts often involve the administration of social activities. One initiative of particular interest, in terms of cultivating social relationships, and therefore enhancing mental health, is one-to-one support that is provided through befriending. Befriending involves matching those with a mental illness with an unpaid, non-professional volunteer; the aim being to foster a companionship which provides reliable and regular support [8].

Certainly, in the realm of mental health, befriending schemes are organised to facilitate social support and social activities; easing the social isolation prevalent among those with a mental illness [8, 18]. Indeed, Hallett et al. [13] noted that befriending schemes most often outlined their key aim as being “patient social and community enhancement”. Thus, the assumption underlying such schemes is that providing this one-to-one support allows these individuals to not only gain a new source of social support but also to further enhance their social networks [25].

Befriending schemes have been shown to have moderate benefits on patient symptoms for various health problems [21]. Specifically, a number of effectively designed studies have explored befriending programmes in relation to individuals with mental illness (e.g. [8, 24, 14, 18]). Whilst findings across these studies vary, they do provide an insight into the effectiveness of befriending particularly in terms of an enhanced social and psychological functioning among those receiving the support of a volunteer [8, 4, 7, 14].

Whilst this previous research has outlined the benefits of befriending, particularly in enhancing the social outcomes of many individuals with a mental illness, there seems to be a lack of focus on the actual befriending pair; that is, the nuances of what occurs in these relationships, how do the individuals perceive each other and what mechanisms are occurring that are possibly enabling, or obstructing, these positive changes mentioned above. Indeed, it is vital to truly understand what it is about befriending that makes it effective from the accounts of not only the befriendee but also the befriender; a stance which only a few have taken as of yet (e.g. [16, 17, 19]).

The present study explored the experiences and motivations of both volunteers and befriendees involved in a number of befriending schemes across the UK. The schemes studied were largely similar in many aspects including how befriendees were referred, how volunteers were recruited and the length of the commitment. There were also similarities in terms of training and supervision requirements, suggesting that the experiences of volunteers and befriendees may be comparable. Semi-structured interviews allowed for a detailed compilation of accounts. We aimed to gain a deeper understanding of the process of befriending through the analysis of the personal experiences recounted by all individuals interviewed.

## Methods

### Aim

To investigate the motivations and experiences of both volunteer befrienders and befriending recipients (hereafter, befriendees) who participate in befriending schemes designed for individuals with mental illness.

### Participants

The study employed a purposive sampling technique so as to recruit individuals from a wide range of services across the UK including eight voluntary sector organisations and four NHS organisations. All participants were referred to the research team by the volunteer coordinator of each organisation. Coordinators were asked to refer people fitting the following inclusion criteria:

*Befriending volunteers* were currently participating or had previously participated in a befriending scheme providing one-to-one input to someone with a mental illness, had not used secondary mental health services in the previous year (as this would fall under the remit of peer support), and they were over the age of 18.

*Befriendees* were over the age of 18, participating in a mental health befriending scheme and were diagnosed with a chronic mental illness (not including: drug or alcohol addiction, eating disorders, dementia, autism syndrome, primary diagnosis of an intellectual disability, ADHD).

### Procedure

The research team contacted volunteer coordinators for each organisation, explained the research to them and asked them to discuss it with the volunteers and befriendees within their scheme. Potential participants, who were interested in taking part, were given contact details of the research team and were sent an information sheet. A face-to-face meeting, either in the participant’s home or at the voluntary organisation premises, was then arranged with those who were eligible and interested in completing an interview. Researchers then explained the participant information sheet with participants and answered any questions. If participants were happy to continue, written consent was taken. Participants were then interviewed by the researcher. Interviews were audio-recorded and transcribed.

### Data collection

Participants answered questions from a semi-structured interview schedule and researchers followed up on interesting points and probed for further information where appropriate. Volunteer befrienders were asked questions on what first motivated them to volunteer, what activities they participated in with their befriendee, what impact the relationship has had on them and what impact they feel it has had on their befriendee, any difficulties they have faced and their experience with the voluntary organisation. Befriendees answered questions on similar topics, including their motivation, the impact the relationship has had on them, their experiences with the voluntary organisations as well as their opinion on what makes a good befriender and how they view the relationship.

### Data analysis

The qualitative data was analysed thematically using the Braun and Clarke [5] guidance, using an inductive approach. In the first stage, two researchers (MC and RT) read and familiarised themselves with 25% of the interview transcripts and independently generated initial codes. They then compared their initial codes and noted where there was strong overlap. An initial set of codes was then agreed upon where both researchers had described a concept in some way, with both researchers agreeing on the code. In the second stage of analysis, the researchers then agreed on coding half of the total data corpus each, such that each researcher would take one section of the coding frame and code all interviews under this frame to ensure consistency. During the coding process the researchers reflected on further elements of the transcripts that may not be captured, discussed possible omissions and added these codes to the framework.

Once all interviews were coded, the researchers then discussed where codes appeared to overlap, consolidated and merged codes where necessary and agreed on the final theme structure on which the results discussed below are based. Data were analysed using NVivo 11.

## Findings

### Participants characteristics

In total, 38 befrienders and 23 befriendees, from eight voluntary sector organisations and four NHS organisations throughout the UK (See Table 1), were interviewed. Demographic information for seven volunteers was not collected. Demographic information for all befriendees and 31 befrienders can be found in Table 2.

**Table 1:** Befriending Schemes

Befriending Scheme	Number of Interviews		
	Volunteers	Befriendee	Total
Mind Doncaster	6	3	9
Hestia Tower Hamlets	3	0	3
Llanelli Mind	5	5	10
East London NHS	2	0	2
Battersea BF Network	6	0	6
Mind Harrow	4	1	5
South West Yorkshire NHS	0	2	2
Together UK	4	4	8
Maidstone Mind	2	2	4
Greater Manchester NHS	5	6	11
Nottinghamshire NHS	2	0	2
TOTAL	38	23	63

**Table 2:** Participant Demographics

	Befriendees (*N* = 23)	Volunteers (*N* = 31)^a^
Male N (%)	11 (47.8)	9 (29.0)
Mean Age	57.7 (22-94 yrs)	42.4 (20-68 yrs)
Employment N (%)		
Employed	1 (4.3)	20 (64.5)
Unemployed	12 (52.2)	6 (19.4)
Retired	9 (39.1)	2 (6.5)
Voluntary work	1 (4.3)	0 (0)
Student	0 (0)	3 (9.7)
Living Situation N (%)		
With partner	2 (8.7)	16 (51.6)
With parents	2 (8.7)	3 (9.7)
With child/ren < 18 yrs	1 (4.3)	4 (12.9)
With child/ren ≥ 18 yrs	2 (8.7)	2 (6.5)
With flatmates	2 (8.7)	3 (9.7)
Live alone	13 (56.5)	3 (9.7)
Primary Diagnosis N (%)		
Mood/anxiety disorder	16 (69.6)	–
Personality disorder	2 (8.7)	–
Psychosis	3 (13.0)	–
Prefer not to say	2 (8.7)	–

^a^Demographic information missing for seven volunteers

Three clear key themes were identified within the data by both researchers: (1) Personal growth & altruism as motivations for volunteering, (2) Benefits for Befriendees through passive and active means and (3) Negotiating between professional role and friendship. Each of these key themes had several related subthemes as shown in Table 3.

**Table 3:** Themes

Main Theme	Sub-themes	
1. Personal Growth & Altruism as Motivators for Volunteering	Personal Growth	Career progression
		Making Amends
		New Experiences
		Feeling Valued
		Enjoyment & Interest
	Altruistic Motivations	Contributing to society
		Previous Mental Health Experience
2. Impact of “doing things” versus “being there”	“Being There”	“Just Chatting”
		New Perspectives
	“Doing Things”	Getting Out
		Giving Advice
1.3. Negotiating Between Professional Role & Friendship	Professional Role	
	Friendship	

### 1. Personal growth and altruism as motivations for volunteering

Volunteers reported a number of motivations for starting volunteering within mental health, as well as motivations for continuing their volunteering role. Motivations for volunteering appeared to vary along a spectrum – from providing them with personal gains to being more altruistic in nature – with many providing several reasons for becoming involved.

### Personal growth

#### Career progression

A number of volunteers cited their main motivation to begin volunteering in mental health, was to gain the experience and skills to help strengthen their chance of a career in mental health, or a similar health care role. They noted that the experience was useful for enhancing their CVs and providing valuable experience for job applications and interviews.

*“Well, at the beginning I got into it because I wanted to gain something for my CV.”*

Volunteer 27

Several volunteers were students or planned to start studies in a health-related field and so considered becoming a befriender for those with mental illness as beneficial in providing them with first-hand experience that they were perhaps unable to obtain through other means.

“I was studying psychology and I wanted experience, so to work with people with mental illness. […] So volunteering is like the best thing to do whilst I was actually studying”

Volunteer 20

The confidence from engaging in something out of their comfort zone helped to enhance their confidence surrounding their abilities; aiding them to become more self-assured in doing something similar again.

*“Having had the experience gave me more confidence that I would be comfortable and happy working with similar populations in future. So that was a positive thing as well.”*

Volunteer 2

### New experiences

A number of befrienders discussed that a desire to meet new people, in addition to having new experiences, was a key motivator for their volunteering. Being able to learn from others and share new experiences with them was important for many volunteers. Some noted how this was especially true when working alongside individuals with mental illness as they often had different insights and personal experiences to share with them.

*“And also, on a personal level, feeling that you are making a connection with someone, I always find that a positive thing, and especially in a challenging circumstance such as someone with a mental health illness.”*

Volunteer 2

For some befrienders, volunteering offered them the opportunity to make social connections in a new city or town where they had few social contacts themselves. They noted that their social networks had grown through volunteering, for example, by meeting other volunteers or the family or friends of their befriendee.

Volunteers also valued the opportunity to do things and go places with their befriendees that they wouldn’t normally. Moreover, volunteers appreciated meeting and socialising with someone they wouldn’t ordinarily meet in their day-to-day life, allowing them to encounter new perspectives and opinions, whether they agreed with them or not.

*“As I say, I think it’s done me good because meeting […] like that expands your horizons, doesn’t it?”*

Volunteer 1

### Feeling valued

A large number of volunteers noted that one of their motivations to continue with befriending was the feeling of being valued and appreciated by their befriendee. Befriendees often demonstrated this through their behaviour as well as explicitly telling their befriender how much they meant to them. This confirmation that what they were doing was meaningful was a strong incentive to continue volunteering and confirmed the value and purpose of their role.

*“She tells me how much she appreciates me all the time, which is nice. She doesn’t need to say that. When I turn up at her house and she spots that it’s me, that face. Sometimes she just throws her arms out and has just got this big smile on her face and then she’s just really excited.”*

Volunteer 28

### Enjoyment and interest

Volunteers reported continuing their befriending relationships because they found their befriendee engaging and interesting; they enjoyed spending time with them. These are the same facets that are important for maintaining any friendship, and this demonstrates the depths of some befriending relationships. In particular, how closely they may come to resemble or even develop into a ‘true’ friendship.

*“Yeah, I enjoyed spending time with her, yeah. I always felt good when I came away from the session.”*

Volunteer 7

### Altruistic motivations

Many volunteers, as well as having personal reasons for befriending also viewed it as an opportunity to “give back” and contribute to society in a positive way; to engage in a selfless act that could ultimately make a difference to the life of someone in need of support, and in turn, the community as a whole.

### Contributing to society

The motivation to “give something back” or “make a difference” was common amongst befrienders for why they began volunteering. By helping someone in need many befrienders believed they could help to improve their community and make it a better place; emphasizing the motivation to volunteer as their duty as members of the community.

*“Sharing some of your time as a gift in some ways. I think, certainly, it’s something that we should all do. Whatever you want to do, I think it would be great to be able to have that as part of society because you can make a difference.”*

Volunteer 12

Two volunteers stated religious or spiritual motivations as their main drive for volunteering. Strong community values associated with the volunteers religious beliefs had initially influenced them to volunteer and to ‘give back’ and to add meaning and purpose to their lives.

*“I suppose I would subscribe to the view, that in relation to my Buddhist practice I need to be doing things that are practical and useful and have some sort of bearing on everyday life.”*

Volunteer 38

### Making amends

A small number of volunteers reported engaging in volunteering as a means of making amends for past behaviour which they perhaps regretted.

*“I just wanted to be good, I felt guilty about things I’d done, I wanted to make reparation. That sort of thing.”*

Volunteer 38

In one case, the befriender was volunteering in mental health as a way to recompense for not having been as supportive of his mother’s mental illness as he could have been. Volunteering with others with mental illness was a means to rectify this.

*“I guess, also for me, though I didn’t like to admit at the time, there’s a little bit of trying to ease my conscience, because my mum didn’t have good mental health, and we didn’t necessarily get on, and I felt guilty that I didn’t do more for her”*

Volunteer 14

### Previous mental health experience

Many befrienders said they had the inclination to volunteer because they had personal experience of mental illness – either having a current or previous mental illness themselves or having a close friend or family member who had a mental illness. For those who had a mental illness themselves they felt that befriending offered them the opportunity to provide support to someone who is in a similar situation to they were – this could either be as a way to give back for those that had helped them through their distress or alternatively to provide the support that they would have appreciated but never received. Those who had family or friends with a mental illness similarly felt that befriending was a way to give back and recompense for those who had helped their friend or family member.

*“I was ill a long time ago – in 1983 I was ill, but I got over it and I recovered fully from my mental health, ill health, and I thought that I wanted to do something to help other people what I had gone through.”*

Volunteer 33

*“Well, I suppose in the past I did have depression myself, and I think I would have valued having someone that I knew I was going to see on a regular basis, that I didn’t have to feel guilty about seeing them.”*

Volunteer 14

A small number of befrienders went as far to say they believed that it was imperative for befrienders in mental health care to have personal experience of mental illness as they may not be able to have a true understanding of what their befriendee was experiencing and how they could provide support.

*“I’ve been very, very ill in my life and it took me to be ill to understand what other people are going through. … I think it’s quite important that you’ve walked the road, you know what it’s like yourself, you know what you would have liked. I would have liked someone like me to come and visit me and take me out and things like that, but it didn’t happen then. It’s too long ago.”*

Volunteer 17

A number of volunteers also had professional experience in mental health care and they believed that the skills they had developed in their previous role were ideal for volunteering with individuals with mental illness.

### 2. Impact of “doing things” versus “being there”

Befriendees reported a range of benefits to their lives as a result of taking part in a befriending scheme and from their interactions with their befriender. Some of these benefits appeared to have resulted from passively being in the company of their befriender – “being there” - whereas other benefits appear to have developed through the befriender’s intentional actions and goals for the relationship – “doing things”. Regardless of what type of befriender they had, all befriendees noted that some aspect or mechanism of their relationship helped to improve their mood and outlook on life.

### “Being there”

#### “Just chatting”

Many befriendees and volunteers refer to their conversations together as largely being non-specific, rather than about any topics in particular; what was described as informal, everyday conversation. Befriendees appreciated having someone to talk to, someone to keep them company and, as many alluded to, simply have a cup of tea with. Indeed, many valued having a talkative and chatty volunteer; particularly in terms of increasing their social skills when they had little to no other friends to converse with.

*“Well, like I say, I don’t have a lot of friends. So engaging with [volunteer], we talk non-stop, really, usually about trivial things, and what have you. It’s just like he’ll start a conversation up about something and then I’ll join in or I’ll start a conversation up and he’ll join in. We get on quite well like that.”*

Befriendee 2

Befriendees emphasized the enjoyment they experienced by simply getting together and talking. The ability to talk freely about numerous matters with their befriender allowed individuals to feel a sense of ease and comfort within their relationship. However, this type of general talking was not always regarded in a positive light and didn’t suit all, with one befriendee conveying the experience as somewhat exhausting.

*“No, it’s just talking, talking, talking. It drains you.”*

Befriendee 8

#### New perspectives

Befriendees often mentioned how spending time with their befriender and listening to their thoughts and opinions as they chatted, had given them a new perspective and outlook on certain things. Often their befrienders were different to the people they would normally encounter and as such they were able to absorb the novel perspectives of their befriender, learn from them, and enjoy their company.

*“But I’m not a person like that; I just look forward to [Befriender] coming. Even though, as I say, I’ve got my own family, it’s different. An outsider is so much different. It’s not that you tell them intimate things, you just have a different outlook to different things to what your family do.”*

Befriendee 14

### “Doing things”

#### Getting out

Comparable to ‘just talking’, a lot of befriendees mentioned how they appreciated just getting out of the house with their befriender. A lot of befriendees noted that before they began their befriending relationship, they wouldn’t leave the house on a regular basis. Having someone come every week or fortnightly to get them out of the house was valued by these individuals, as it relieved boredom, ensured they did some exercise and gave them something to look forward to.

*“Just being out there really. Just being out and just doing stuff. I wouldn’t do it if it were me on my own, because, now, whether I’m too cautious or too nervous or whatever. But yeah, just having him there and being able to just say, ‘Come on, we’ll go and have a look down here,’ and do it without worrying, that’s just the best thing really.”*

Befriendee 13

In turn, several befriendees discussed how they were now doing things on their own which they wouldn’t have done previous to their befriending relationship. Simply by going out with their befriender, they felt this had increased their confidence to socialise more and as such, become more independent. This increase in independence is importance as it could potentially lead to engaging in their community more and potentially helping to reduce any social isolation experienced.

*“Yeah cause I’ve increased my self-confidence to a certain level I mean I can go around by myself whereas before I wasn’t able to and stuff like that, but it’s an on-going progression.”*

Befriendee 1

#### Giving advice

The majority of volunteers experienced situations where their befriendee would turn to them for advice and encouragement on a range of topics, including jobs and relationships, as opposite to general small talk (see ‘just chatting’). Some befrienders felt comfortable giving them their perspective on problems and even offering advice, as best they could.

*“Sometimes she’d seem a bit anxious if something had happened with a friend or girlfriend, or something, and she’d be stressing out about that. […]I was just trying to offer her a bit of relationship advice and things… But yeah, just, kind of, talked through things with her a little bit really and put things into perspective.”*

Volunteer 7

However, a number of volunteers seemed cautious in regard to giving advice; avoiding telling their befriendee what to do in a given situation. Indeed, there was awareness amongst some befrienders that they should not be counselling their befriendee or assuming the role of a therapist.

*“Sometimes I feel, also, a bit helpless because I’m a volunteer, I haven't really had the proper training, I’m not a psychologist, and I’ve got a feeling he wants to get advice from me.”*

Volunteer 3

Befriendees also reported developing certain coping strategies due to their relationship with their befriender; becoming better able to handle feelings of anger, stress or anxiety associated with their mental illness. Befriendees implied that this was thanks to their befriender talking them through their worries or problems and helping them to organise their thoughts.

*“The one thing I didn’t say in the first bit…is that it’s helped me in the sense that I’m not as aggressive as I used to be…”*

Befriendee 20

### 3. Negotiating between professional role and friendship

The befriender role, what it meant to individuals and what it entailed was a contentious issue amongst participants, especially the befrienders themselves. Like all relationships, the befriending relationship is complex with volunteers experiencing several challenges that they had to navigate and address throughout their time together. Unlike the majority of other relationships people have, however, there is the added complexity of a third party – the voluntary organisation – forming and facilitating the relationship. Often volunteers had to negotiate between what the voluntary organisation recommended or required of them, what they believed was appropriate and the expectations of their befriendee. This led to many volunteers describing their relationship with their befriendee along a spectrum – from akin to a professional role to being more like a ‘true’ friendship.

#### Professional role

For volunteers who saw themselves as closer to a professional, boundary setting was approached more diligently. Often, they did not deviate from the boundaries that the voluntary organisation had set down; for example, not disclosing their personal phone number in addition to emphasizing when and how often their befriendee could contact them. In turn, those that exercised more boundaries in the relationship often chose not to disclose any personal information about themselves which meant that conversations were often one-sided, with the befriender taking on a listening role, rather than reciprocating and sharing their personal experiences.

*“Much closer to a professional relationship. […] Um, so um, I would see it as much closer to that. I don’t see it as a friendship which is equal. I see myself as mainly, eh, there to provide what that person wants. Now that can be, now there are problems with that but I still feel there are boundaries which I have to follow.”*

Volunteer 37

When questioned about the ending of the relationship, there was again a lot of variation in what was considered the ‘right’ time to end the relationship. For those who saw themselves as more professional, they often ended the relationship by what was prescribed by the voluntary organisation, which was normally a year. For organisations that did not prescribe a length it was often up to the three parties to decide between themselves when the relationship should end and this was loosely defined as when the befriendee no longer ‘needed’ the support of a befriender.

#### Friendship

Some volunteers took a more relaxed approach to their role and saw it more akin to a ‘true’ friendship, albeit one that has input from the voluntary organisation. These volunteers sometimes begin their role adhering to the boundaries that the voluntary organisation had prescribed, but as the relationship developed they felt some of these boundaries were too strict and got in the way of developing a more reciprocal and balanced relationship. They felt that finding a compromise between what the voluntary organisation wanted and what the befriendee wanted was the best way to build a trusting and more equal relationship. Often it was the case that boundaries set at the beginning of the relationship tended to evolve and adapt to suit the needs of the specific pair as their relationship developed over time.

*“No. Didn’t bother, because I thought that would be too restrictive. […] What grounds do you set in a relationship? Do you set grounds? They evolve, don’t they? If you were meeting another colleague at work, there are colleague ground rules. Then all of a sudden you might like that colleague and therefore you go out with her for a drink at night, the ground rules change. They change[…] Life just changes with it. You can’t write ground rules.”*

Befriendee 20

When it came to ending the relationship, those who saw their befriendee as more like a friend, had issues with how they would end the relationship. Several chose to continue seeing or communicating with their befriendee after the prescribed time set by the voluntary organisation as they didn’t want to stop being friends with their befriendee entirely. This often meant that they would still contact them or meet up with them, but on a less regular basis and without the continued support of the organisation.

*“As I say, because you know that it’s going to end at some point and it’s a service. Which doesn’t underline most friendships. You are not thinking, well, in six months’ time I won’t know this person anymore. So it’s quite difficult.”*

Volunteer 1

## Discussion

Three broad themes were drawn from the data to describe the experiences of both volunteer befrienders and those receiving befriending in mental health care. The first theme details the volunteers motivations to become a befriender, and in particular the motivation to volunteer with people with mental illness. These motivations ranged from being altruistic to reasons of personal growth. It was common for volunteers to offer both personal and altruistic motivations when asked why they started befriending with a mental health population. The second theme revealed the impact the befriending relationship had on the befriendees. They discussed benefits that the relationship afforded them which depended on the particular type of the relationship they had; whether the befriender was there to listen to them and simply provide company (“Being there”) or if they were more focused on getting them out of the house and being more activity-focused (“Doing things”). Lastly, volunteers discussed at length the complexity and nuance of their role as befriender. This theme dealt with how befrienders negotiated between being like a friend or a professional, and how their expectations, those of the volunteering organisation and their befriendee influenced this. This, in turn, reflected what boundaries they did or did not maintain within the relationship.

### Strengths and limitations

In this study we explored the views and experiences of volunteers who worked directly with people with mental illness in a befriending context as well as the befriendees themselves. One of the main strengths is that participants were sourced from twelve different befriending schemes, including both NHS schemes and from the voluntary sector. The themes described above are therefore widespread across the schemes and show consistency in the experience of participants of befriending in mental health care. Furthermore, our findings are consistent with previous literature on volunteering and specifically befriending, both in mental health care and other areas of focus. One limitation of the study is that the length of the volunteer-befriendee relationship was not recorded. It is conceivable that the relationship length could have had either a positive or negative impact on experience. This could have been experienced in various forms, such as how the relationship affected the lives of both parties, or the nature of the activities undertaken as a pair. A further limitation of the study is that volunteers and befriendees were referred to the research team by the volunteer coordinator of each befriending scheme. Participants within this study reported mostly positive experiences of their voluntary activity and it is possible that volunteers known to be enthusiastic to the volunteer co-ordinators were disproportionately referred to the research team and perhaps those who had less positive experiences were not included in the sample. To help account for this, the questions in the interview schedule aimed to address the potential negative aspects of befriending as well as positive ones (for example regarding possible stressed, conflicts, and what didn’t work). Although volunteer co-ordinators referred participants to this study, the views of the co-ordinators themselves regarding the motivations and experiences of befriending were not addressed. Therefore this could be an area of interest for future research. Furthermore, future research could consider whether the motivations and experiences of volunteers and befriendees differ according to geographical location and duration of the befriending relationship.

### Comparison with literature

#### Personal growth and altruism as motivations for volunteering

Given that volunteers are unpaid and have other constraints on their time, being aware of why people volunteer is vital for their retention in voluntary organisations. In line with the present study, Proteau and Wolff [20] believed people’s reasons for volunteering could be explained by three models: 1) to enhance employment opportunities, 2) to enhance other’s well-being and, 3) for personal growth or gain. Gidron [12] also described volunteer motivations to be a combination of ‘social’, ‘personal’ or ‘indirectly economic’. Although this literature isn’t specifically in mental health care, the similarities with the current study are evident. Hallett et al. [13], who looked at volunteering for people with severe mental illness, also grouped reasons for volunteering into two broad categories – ‘giving’ and ‘getting’. We found that motivations for personal growth and gains were most commonly reported, whether it is for enhancing career-related experience and skills, increasing opportunities for new experiences or to make amends and make one feel better. Klug et al. [15], also found that the top five most common reasons for befriending, in a sample of 360 mental-health befrienders, related to either doing something for others – “give something back”, “feel a responsibility to help others”, “helping others is part of my philosophy of life” – or to achieve something themselves – “enhance my awareness of mental health issues”, “acquire new skills”. Although many volunteers reported an altruistic reason as their primary motivation to start befriending, they often reported additional personal reasons as well.

Previous mental health experience, although not given as a motivation as often as personal reasons, seemed to be a very important motivation for those who did report it. From the perspective of potential befriending receivers, Toner et al. [26] found that in a sample of individuals with serious mental illness the majority said they would prefer a befriender who had “lived experience as a patient in mental health care”. Lived experience was also highlighted as a strong factor in volunteering in Aguirre and Bolton’s [1] meta-synthesis of the motivations of crisis volunteers and as they highlighted, this is important for voluntary organisations to ensure that those volunteers are ‘ready’ for the role and can be utilised effectively for the experience they have.

#### Impact of “doing things” versus “being there”

Although volunteer befrienders have been found to benefit from their befriending relationship [13, 19] the impetus for orchestrating a befriending relationship is to support, in some way, an individual who is in need. Through our interviews both volunteers and befriendees noted numerous benefits that they had perceived or experienced, respectively. We found that these personal gains were often linked to the type of relationship that formed between the pair. Some volunteers assumed a more passive role of just ‘being there’. These benefits were gained through simply being in the company of another person on a regular basis, having a chat and learning from them. Some volunteers noted that they took on a more active role and had a loosely formed goal for what they wanted to achieve with their befriendee, e.g. to get them out of the house more. Both of these approaches were appreciated by the befriendees and highlights the various ways a befriending relationship can impact on a person’s life. Dodd et al. [10], whilst looking at befriending in end-of-life care also made the distinction between ‘being there’ and ‘doing for’ and how adopting either of these roles may impact differently on their befriendee. In a mental health sample, Toner et al. [26] found that that patients, when asked what their befriender would be like, the majority preferred someone who took a more passive role, compared to someone ‘who does activities with me’. However, when asked specifically to think about activities with this befriender, 61% they would want them for ‘just getting out of the house’.

### Negotiating between professional role and friendship

A theme that was discussed by all of the volunteers was the complexity of their role and the ambiguities that surrounded what was expected of them and what boundaries were required for the relationship. This theme is also important for retention of volunteers because it is likely that if they are worried, uncertain or stressed about what they are doing, they could stop befriending altogether. Importantly, Yanay and Yanay [27] found that volunteers in crisis-related roles were demotivated when they found a lack of direction from the voluntary organisation as well as lack of support in fulfilling their roles. This finding was supported by qualitative findings from Aguirre and Bolton [1] who found uncertainty about their role and lack of guidelines to be an issue. From our findings this ambiguity largely centred on whether the befriender was meant to be more like a ‘true friend’ or to act as a pseudo-professional. Volunteers often mentioned that the organisation may have set certain boundaries on the relationship (e.g. not sharing personal information), but some volunteers found these rules hard to implement when they were face-to-face with their befriendee. This meant that some volunteers had few boundaries with their befriendee, acting like a ‘true friend’, some maintained strict personal boundaries and were seen as and saw themselves as similar to professionals and many were somewhere along this spectrum, having some boundaries but discarding others they didn’t feel were conducive to building a trusting relationship. This relationship spectrum was also documented by Thompson et al. (2015), in their conceptual review on the meaning of befriending in mental health care. The review highlighted several risks and benefits for each type of role. For a more professional role there are clearer goals and boundaries, however, there is the risk the befriender may act like a therapist, without the proper training to do so. For more friend-like relationships there is no pressure ‘to achieve something’ and the befriendee feels valued as an individual, however, there is the associated risk of emotional turbulence that comes with a ‘real’ friendship. They surmised that it is important to the organisation to be aware of the risks that could be associated with assuming either type of role but that different approaches may be appropriate for different contexts and certain individuals. As such voluntary organisations should query both potential volunteers and potential befriending recipients as to what they would ideally like from their relationship. In Toner et al.’s [26] study, they found that when individuals with SMI were asked what their preferred befriending relationship would be like, the majority said they would want ‘a real friendship’. This again is echoed in McCorkle et al. [17] who found that individuals with SMI in a compeer relationship (a form of befriending relationship [22] believed a strong match was ideally with someone with “no particular agenda other than to be social”.

### Implications of findings

Understanding the motivations of those who volunteer in mental health care, as well as how they and their befriendees perceive the role is important for voluntary organisations and coordinators, as it can help to optimise and fully utilise the invaluable resource that volunteers provide. It can also guide coordinators on how to best match befriending pairs based on their respective motivations and what they want to gain from the relationship. When recruiting volunteers it may prove beneficial to note their motivations as this could potentially impact the ‘type’ of befriender they become or how long they intend to volunteer for. Even those that intend to volunteer for a short period to boost their CV are valuable, since many befriendees may only need short-term befriending support. The ambiguity around the role is something that could be addressed in the training and supervision of volunteers, making the role very clear and offering support to volunteers (and their befriendees) if the relationship develops into something that didn’t meet their initial expectations. Establishing what befriendees want to get out of their befriending experience is also important as some preferred just having someone to talk to and didn’t particularly want to do activities, whereas other valued having the support and company to leave the house. Knowing their preference for this could help when matching them to volunteers who would be best suited to the tasks, e.g. someone who is particularly adept or interested in at listening or conversation, as opposed to simply matching based on socio-demographic characteristics.

The results from this study indicate that there is no ‘textbook’ method to manage and coordinate befriending relationships. As such, voluntary organisations may benefit from taking individual needs into account when matching and supporting befriending pairs.

Although volunteer co-ordinators referred participants to this study, the views of the co-ordinators themselves regarding the motivations and experiences of befriending were not addressed.

## Conclusion

This study explored the experiences of individuals of befriending relationships in mental health care. The findings were consistent with those found in other studies looking at befriending relationships in other areas. Notably, individuals chose to befriend for a variety of reasons - both personal and altruistic in nature; those who receive befriending report varying experiences from the relationship and that the role of the befriender is incredibly nuanced and sometimes challenging for the befriender to negotiate. This suggests that there are various models of befriending relationships that work best for different people and as such there is no ‘one-size-fits-all’ way to manage and organise a befriending scheme and no ideal type of befriending relationship.

## Abbreviations

ADHD: Attention Deficit Hyperactivity Disorder
CV: Curriculum Vitae
NHS: National Health Service
UK: United Kingdom

## References

[1] Aguirre RTP, Bolton MW (2013). Why do they do it? A qualitative interpretive meta-synthesis of crisis volunteers’ motivations. Soc Work Res.

[2] Angell B (2003). Contexts of social relationship development among assertive tradition and modernization. Br J Psychiatry.

[3] Baker F, Jodrey D, Intagliata J, Straus H (1993). Community support services and functioning of the seriously mentally ill. Community Ment Health J.

[4] Bradshaw T, Haddock G (1998). Is befriending by trained volunteers of value to people suffering from long-term mental illness?. J Adv Nurs.

[5] Braun V, Clarke V (2006). Using thematic analysis in psychology. Qual Res Psychol.

[6] Dailey WF, Chinman MJ, Davidson L, Garner L, Vavrousek-Jakuba E, Essock S, Marcus K, Tebes JK (2000). How are we doing? A statewide survey of community adjustment among people with serious mental illness receiving intensive outpatient services. Community Ment Health J.

[7] Davidson L, Haglund KE, Stayner DA, Rakfeldt J, Chinman MJ, Tebes JK (2001). “It was just realizing…that life isn’t one big horror”: a qualitative study of supported socialization. Psychiatric Rehabilitation Journal.

[8] Davidson L, Shahar G, Stayner DA, Chinman MJ, Rakfeldt J, Tebes JK (2004). Supported socialization for people with psychiatric disabilities: lessons from a randomized controlled trial. J Commun Psychol.

[9] Davidson L, Stayner D (1997). Loss, loneliness, and the desire for love: perspectives on the social lives of people with schizophrenia. Psychiatric Rehabilitation Journal.

[10] Dodd Steven, Hill Matt, Ockenden Nick, Algorta Guillermo Perez, Payne Sheila, Preston Nancy, Walshe Catherine (2018). ‘Being with’ or ‘doing for’? How the role of an end-of-life volunteer befriender can impact patient wellbeing: interviews from a multiple qualitative case study (ELSA). Supportive Care in Cancer.

[11] Giacco D, McCabe R, Kallert T, Hansson L, Fiorillo A, Priebe S (2012). Friends and symptom dimensions in patients with psychosis: a pooled analysis. PLoS One.

[12] Gidron B (1978). Volunteer work and its rewards. Volunteer Administration.

[13] Hallett C, Klug G, Lauber C, Priebe S (2012). Volunteering in the care of people with severe mental illness: a systematic review. BMC Psychiatry.

[14] Harris T, Brown GW, Robinson R (1999). Befriending as an intervention for chronic depression among women in an inner city. 1: randomised controlled trial. Br J Psychiatry.

[15] Klug Gϋnter, Toner Sarah, Fabisch Karin, Priebe Stefan (2018). Characteristics and motivations of volunteers providing one-to-one support for people with mental illness: a survey in Austria. Social Psychiatry and Psychiatric Epidemiology.

[16] Lieberman AA, Gowdy EA, Knutson LC (1991). The mental health outreach project: a case study in self help. Psychosocial Rehabilitation Journal.

[17] McCorkle BH, Dunn EC, Wan YMW, Gagne CD (2009). Compeer friends: a qualitative study of a volunteer friendship programme for people with serious mental illness. Int J Soc Psychiatry.

[18] McCorkle BH, Rogers ES, Dunn EC, Lyass A, Wan YM (2008). Increasing social support for individuals with serious mental illness: evaluating the compeer model of intentional friendship. Community Ment Health J.

[19] Mitchell G, Pistrang N (2011). Befriending for mental health problems: process of helping. Psychology and Psychotherapy.

[20] Proteau L, Wolff F (2008). On the relational motive for volunteer work. J Econ Psychol.

[21] Siette J, Cassidy M, Priebe S (2017). Effectiveness of befriending interventions: a systematic review and meta-analysis. BMJ Open.

[22] Skirboll BW (1994). The compeer model: client rehabilitation and economic benefits. Psychosocial Rehabilitation Journal.

[23] Spaniol L, Gagne C, Koehler M (1999). Recovery for serious mental illness: what it is and how to support people in their recovery. The psychological and social impact of disability.

[24] Staeheli M, Stayner D, Davidson L (2004). Pathways to friendship in the lives of people with psychosis: incorporating narrative into experimental research. J Phenomenol Psychol.

[25] Thompson R, Valenti E, Siette J, Priebe S (2016). To befriend or to be a friend: a systematic review of the meaning and practice of “befriending” in mental health care. J Ment Health.

[26] Toner S, Cassidy M, Chevalier A, Leverton M, Pinto da Costa M, Priebe S (2018). Preferences for befriending schemes: a survey of patients with severe mental illness. BMC Psychiatry.

[27] Yanay GV, Yanay N (2008). The decline of motivation?: from commitment to dropping out of volunteering. Nonprofit management and Leadership.

